# Research on the Species Difference of the Hepatotoxicity of Medicine Based on Transcriptome

**DOI:** 10.3389/fphar.2021.647084

**Published:** 2021-04-23

**Authors:** Ziying Xu, Qianjun Kang, Zihui Yu, Lichun Tian, Jingxuan Zhang, Ting Wang

**Affiliations:** ^1^ Beijing Research Institute of Chinese Medicine, Beijing University of Chinese Medicine, Beijing, China; ^2^ China National Center for Bioinformation, Beijing, China; ^3^ University of Chinese Academy of Sciences, Beijing, China; ^4^ Key Laboratory of Genomic and Precision Medicine, Beijing Institute of Genomics, Chinese Academy of Sciences, Beijing, China

**Keywords:** hepatotoxicity, drug induced liver injury, species difference, oxidative stress, steatosis

## Abstract

In recent years, several drugs have been withdrawn from use by regulatory bodies owing to hepatotoxicity; therefore, studies on drug-induced liver injury (DILI) are being actively pursued. Most studies evaluating DILI use rats or mice as animal models to determine drug toxicity; however, the toxicity of a drug can vary between rats or mice. These inconsistencies in *in vivo* studies among different animal models affect the extrapolation of experimental results to humans. Thus, it is particularly important to choose the most suitable animal model to determine drug hepatotoxicity owing to the genomic differences between rats and mice resulting from evolution. In this study, genome-wide transcriptome analysis was used to explore hepatotoxicity caused by differences in species. Our findings provide the preclinical basis to further study the mechanisms of drug hepatotoxicity and aid in the selection of animal models to determine drug safety. We used murine models (Sprague-Dawley and Wistar rats, ICR and Kunming mice) in this study and by using transcriptome sequencing with the differentially expressed genes in rat and mouse livers as the entry point, we explored the mechanism of oxidative stress and the difference in gene expression in the lipid-metabolism pathway between rats and mice. The clinically established hepatotoxic drugs, fructus psoraleae and acetaminophen were used to validate our study. Using pathological studies, we confirmed that oxidative stress in mice was more serious than that in rats, and that Kunming mice were more suited for the study of oxidative stress-related DILI. The validity of our findings was further verified based on gene expression. Thus, our study could serve as a valuable reference for the evaluation of potential preclinical hepatotoxicity. Moreover, it could be used in the prediction and early diagnosis of drug-induced liver injury caused by traditional Chinese medicine or synthetic drugs, thereby providing a new avenue for drug-toxicity studies.

## Introduction

Drug-induced liver injury (DILI) refers to liver injury caused by a drug or its metabolites, or due to hypersensitivity of the special population or tolerance of the liver to drugs, also known as drug-induced liver disease. DILI manifests clinically as various acute or chronic liver diseases. This condition is self-limiting and mild, especially when the drug is discontinued; however, in severe cases, it may result in fatalities (Chalasani et al., 2014; Wang et al., 2018). Currently, more than 30,000 drugs and healthcare products are available, of which more than 1,000 drugs have already been identified to cause DILI (Yu et al., 2015). Therefore, DILI is a serious public health problem that cannot be ignored (Liu et al., 2019). Accordingly, evaluation of drug safety is crucial for drug developers and clinical pharmacists. DILI can result in hepatocyte injury, mixed injury, and cholestatic injury (Wang and Yu 2014; Shen et al., 2019). Therefore, evaluating and predicting the drugs that are capable of causing DILI is often a challenge, as this parameter is an inevitable requirement for the development of translational toxicology and precision medicine.

Owing to ethical and moral aspects, it is not possible to enroll human subjects for an in-depth study of DILI. Therefore, most toxicity data related to new drugs are mostly generated from *in vivo* studies. Currently, rats are routinely used for the preclinical safety evaluation of drugs and toxicity studies; however, the murine model is not suitable for toxicity determination of certain drugs. For example, previous studies have shown that the rat model is clinically relevant for acetaminophen (APAP)-induced hepatotoxicity (McGill et al., 2012). KYang et al. (Yang et al., 2014) found that higher basal transport rate significantly prevented the accumulation of intracellular taurocholic acid (TC) in rats upon the inhibition of bile salt output pump (BSEP), whereas human hepatocytes were more sensitive to the inhibition of BSEP, resulting in a significant increase in intracellular TC levels. This difference may be the reason why many drugs that cause cholestasis in humans do not cause hepatotoxicity in rats. Moreover, Straniero et al. (Straniero et al., 2020) have reported that mice and rats have a better ability to synthesize bile acid (BA) and cholesterol, and exhibit low levels of low density lipoproteins (LDLs) and have a rapid turnover compared to humans. These differences are related to the abundant hydrophilic 6-hydroxyphenylpropionate in normal mice. A suitable animal model would be one that has a metabolic or biotransformation pathway similar to that of humans. Accordingly, the animal model can be successfully used to predict and obtain data and to extrapolate the findings of drug toxicity in humans. Furthermore, for the optimal use of experimental data obtained using animal models and to extrapolate these findings in humans, it is necessary to study the genetic expression, differences in species, the role of gender, and the effects of different drugs between humans and animals. Therefore, it will be of great benefit to study heterogeneous liver injury in humans with a focus on the research methods and species differences in drug-induced hepatotoxicity. Species differences: The genetic composition and expression among different species of mammals differ right from the stage of embryonic development (Kalinka and Tomancak 2012; Abzhanov 2013). These differences lead to diverse pathways of drug metabolism; moreover, different species may have completely different metabolic pathways for the same drug. Additionally, certain physiological or toxic effects may manifest in one species and not in others, and the duration or intensity of drug action among different species may also vary.

High-throughput sequencing has promoted the development of systems-biology research, including genomics and transcriptomics. Analysis of the transcriptome can help determine and quantify changes in the expression level of the transcript in each state (Wang, Gerstein, and Snyder 2009; Costa et al., 2010), even at the single-cell level (Zheng et al., 2018). Thus, owing to its extensive use in the life sciences, transcriptomics is a rapidly evolving field. As the demand for processing research samples is constantly changing, new transcriptome technologies, such as single-strand sequencing and strand-specific sequencing have been developed. These novel transcriptomic techniques require low sample volumes and yield more precise results (Croucher et al., 2009). In disease research, RNA-seq can help researchers understand the pathogenesis of diseases more accurately and shed light on the relationship between specific RNA and diseases, based on the precise regulation of each gene in diseases. Thus, this technology is useful in the development of new drugs and could be valuable in the prevention and treatment of tumors (Ren et al., 2013). Previous studies have attempted to differentiate between cancers in mice and humans, using comparative genomics, gene expression profiling, and proteomic analysis (Touw and Erkeland 2007; Ray et al., 2018). Therefore, cross-species comparison of the transcriptome, tissue-specific methylation levels (Zhou et al., 2017), and gene regulation at the molecular level can not only help determine the differences at the gene-expression level, but also aid in further understanding their similarities and evolutionary correlation through homologous genes (Breschi, Gingeras, and Guigo 2017).

Drug efficacy and toxicity presentations vary among species and have attracted increasing research attention. Understanding the differences in drug toxicity among species can help better interpret results from *in vivo* studies, lead to more precise conclusions, elucidate differences between people, and determine drug efficacy and safety. Differences among species can be attributed to the difference in gene expression and the difference in cellular differentiation during biological evolution. In our previous study on the potential hepatotoxicity of Chinese medicines, we found that the hepatotoxic phenotypes were not the same in rats and mice, and exhibited significant species difference. This finding raised the question of which animal model is more suitable to study potential hepatotoxicity. Accordingly, in our current study, we commenced with the transcriptomics studies of normal liver tissues of commonly used animal models. Based on the analysis of the correlation of gene-expression profiles of the liver in two species of rats and mice, and human, the relevant biological pathways of hepatotoxicity and the differences in pathways among species were studied to identify suitable animal models to study the different phenotypes of hepatotoxicity. Our findings can serve as a reference for the preclinical evaluation of potential hepatotoxicity. Moreover, our study findings could be used for the prediction and early diagnosis of DILI and provide suitable research ideas in the field of drug toxicity.

## Materials and Methods

### Animals and Experimental Design

Eight-week-old male and female ICR and Kunming mice (30 ± 2 g), and 8-week-old male and female Sprague-Dawley and Wistar rats (250 ± 10 g) were purchased from the Beijing Vital River Laboratory Animal Technology Co. Ltd (Beijing, China). The animals were housed in groups of matched weights in a humidity (40–70%)-and temperature (20–25°C)-controlled room and subjected to a 12-h light/dark cycle during studies. The animals were provided access to food and water *ad libitum*. This study was approved by the Experimental Animal Ethics Subcommittee of Beijing University of Chinese Medicine (BUCM-4-2019091801-3067). The study protocols were in accordance with the National Institute of Health Guide for the Care and Use of Laboratory Animals.

For RNA-seq and follow-up experiments, a total of 16 animals, including male and female ICR and Kunming mice, Sprague-Dawley rats, and Wistar rats (two animals of each sex), were used.

Rats and mice were weighed and randomly divided into two groups (control and fructus psoraleae-treated groups), with 10 males and 10 females in each group. The control group was orally administered distilled water, while the treated groups were administered 20 g/day (converted from clinical human dose) of fructus psoraleae daily for one month.

For the APAP experiment, ICR and Kunming mice were weighed and randomly divided into two groups (control and APAP-treated groups), with 6 males and 6 females in each group. The control group was orally administered distilled water, while the treated groups were administered APAP at doses of 400 mg/kg/day for 7 days.

### Histopathological Analysis

After drug administration, the animals were sacrificed humanely. Liver and brain weights were measured. The ratio of each organ weight to the terminal body weight in grams/100 g body weight (relative organ weight) was calculated. The livers were fixed in 10% neutral buffer formalin and embedded in paraffin. Subsequently, the paraffin-embedded samples were sliced into 3-μm-thick sections using a rotary microtome. Next, the sections were stained with hematoxylin-eosin (H&E) and the slides were observed using optical microscopy at ×200 magnification (Carl Zeiss Meditec AG, DE).

### Determination of Enzyme Activity and GSH Levels in Liver

Cellular Glutathione peroxidase Assay kit with NADPH, Total Superoxide dismutase Assay kit with WST-8, catalase Assay kit, and the GSH and GSSG Assay kits were used, following the respective manufacturer’s protocols (Beyotime Institute of Biotechnology, Nanjing, China). Absorbance was measured using a microplate reader (BioTek Instruments, Inc., Winooski, VT, United States). The data are expressed as the ratio of the OD value of the treated group to that of the control group.

### RNA Extraction and RNA-seq

Liver tissues were immersed in liquid nitrogen and the total RNA was extracted using Trizol Reagent (Invitrogen, Thermo Fisher Scientific, Inc., NY, United States) according to the manufacturer’s instructions. A Qubit Fluorometer (Invitrogen, Thermo Fisher Scientific, Inc., NY, United States) was used for RNA determination and quantification. The Ribo-off rRNA Depletion Kit (Vazyme, Nanjing, China) and VAHTS Universal V6 RNA-seq Library Preparation Kit (Vazyme, Nanjing, China) were used to construct stranded RNA-seq libraries according to the manufacturer’s instructions.

### Real-Time RT-PCR

After RNA quantification, equivalent amounts of RNA were reverse transcribed using HiScript II Q Select RT SuperMix for qPCR (+gDNA wiper) (Vazyme, Nanjing, China). Real-time quantitative PCR was performed using ChamQTMSYBR qPCR Master Mix (Vazyme, Nanjing, China) and detected using a BIO-RAD CFX96 Touch system. Gene-expression analysis was performed using the comparative ∆∆CT method with GAPDH for normalization. The primers that were used are as follows:

### RNA-seq and Gene Set Enrichment Analysis

We downloaded the human liver transcriptome data Array Express with the accession code e-MTAB-6814. In the BAM files of the three young adults, the same HTSeq Python package was used to change the expression value into counts. Next, the downloaded homologous genes were used to combine the count value of each sample. The expression levels were determined as counts per million (CPM) using edgeR package, and ComBat was used to eliminate the batch effect between species. Mice and rat transcriptome data were obtained from self-constructed sequencing data, as mentioned in the “animals and experimental design” section. Two biological replicates were performed for each treatment for each sex. Trimomatic (version 0.36) was used and readings with lengths less than 35 nucleotides or those containing ambiguous nucleotides were discarded. The remaining readings were consistent with that of the human reference genome (hg19) and mouse reference genome (mm10) using HISAT2. For each sample, only unique map reads with map quality score ≥ 20 were reserved for subsequent analyses. HT Seq *Python* package (version 0.9.1) was used to count the number of reads of a unique map for each gene. Gene expression was quantified as fragments per kilobase per million (FPKM) and used for correlation and in principal components analysis (PCA). R-package DEseq2 was used to determine the differentially expressed genes between the treatment and control groups; fold-change cutoff = 1.0, *p* value cut off = 5 × 10^−2^. R-package of maSigPro was used to analyze the time series and determine the differentially expressed genes with the duration of drug therapy, Q = 0.05, MT. adjust = “BH,” alfa = 0.05. R-package Cluster Profiler was used to analyze Gene Ontology (GO) of genes for functional enrichment analysis, and only significantly enriched GO terms with *p* < 0.05 were retained. The oxidative stress gene set GO:0006979 and the hepatic steatosis gene set were downloaded from Human Phenotype Ontology, HP_0001397.

### Statistical Analysis

Results are presented as mean ± SD. Results between multiple groups were analyzed using one-way analysis of variance (ANOVA) in SPSS (version 20.0) statistical analysis program, and the differences among means were analyzed using Dunnett’s multiple comparisons test or post-hoc analysis. Differences were considered significant at *p* < 0.05.

## Results

### Homologous Genes in Liver Tissues of Different Strains of Mice and Rats are Mainly Related to Hepatic Function

**FIGURE 1:**
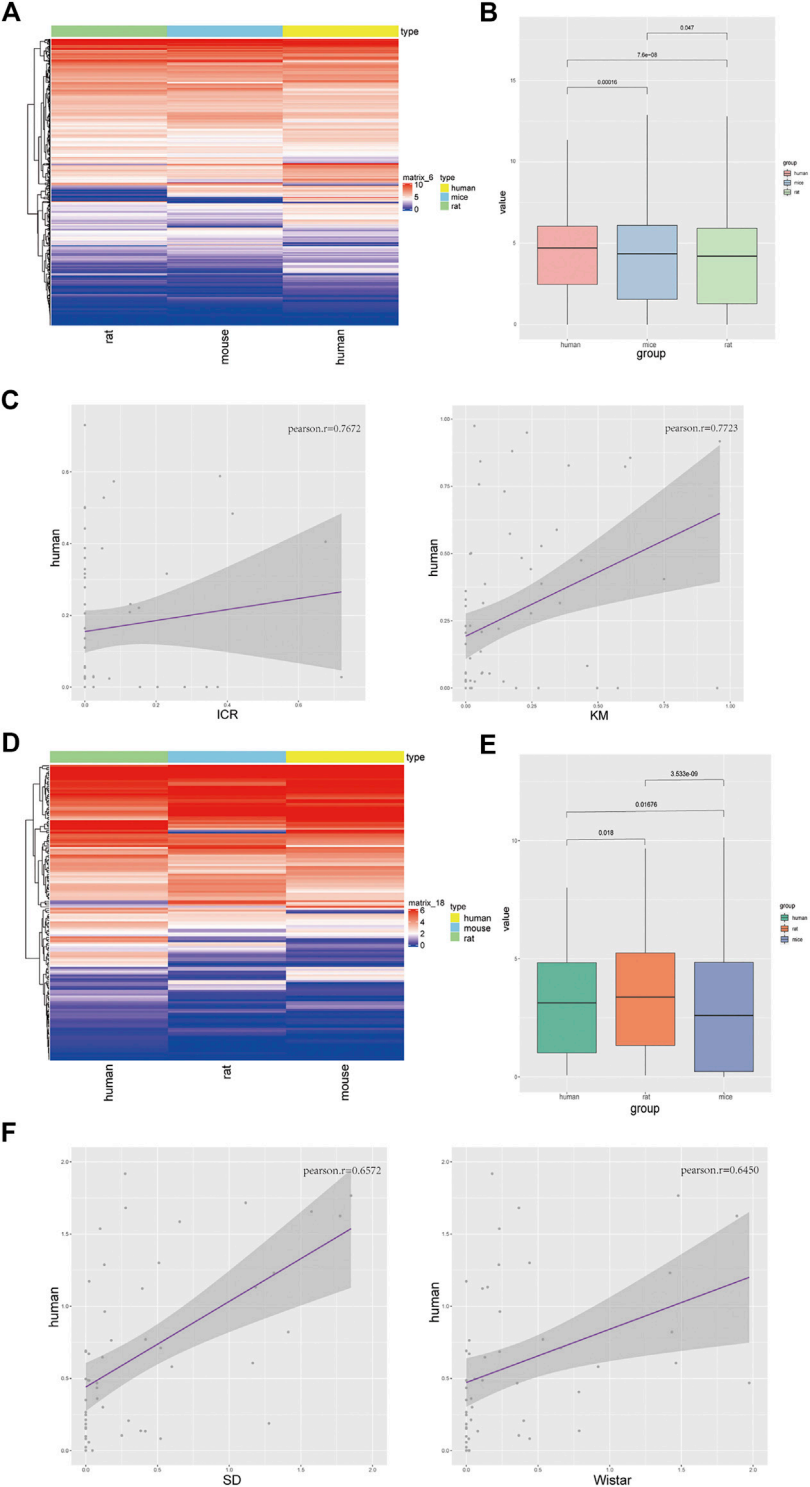
Homologous genes in liver tissues of different strains of mice and rats are mainly related to hepatic function. **(A)** PCA profile of homologous gene expression in mice and rats; **(B)** Correlation and clustering of homologous gene expression profiles of mice and rats **(** Supplementary Table S1 **)**; **(C)** Volcanic map showing the differentially expressed genes of ICR and Kunming mice **(left)**, KEGG pathways of co-expressed genes **(right)**; **(D)** Volcanic map showing the differentially expressed genes of Sprague-Dawley (SD) and Wistar (WS) rats **(left)**, KEGG pathways of co-expressed genes **(right)**.

Total RNAs of liver tissue from male and female Sprague-Dawley and Wistar rats, and ICR and Kunming mice were prepared and transcriptomic sequencing analysis was performed for the removal of ribosomal RNAs. We used two individuals of each sex from each strain. We selected the homologous genes in the dataset of rats and mice for PCA (Figure 1A), cluster analysis (Figure 1B; Supplementary Figure S1A), and gene list of common genes (Supplementary Table S1), and found that the homologous genes of liver tissue in mice and rats were significantly different. However, there were no significant differences between gender (Supplementary Figure S1B). Therefore, the choice between using the rat or the mouse model should be given due consideration when selecting an animal model. After comparing the two strains of mice, the common genes were mainly enriched in the pathways of organic acid catabolic process, carboxylic acid catabolic process, small-molecule catabolic process, fatty-acid metabolic process, and the steroid metabolic process. All these pathways are related to hepatic function (Figure 1C). In rats, we found that the co-expressed genes were mainly enriched in the fatty-acid metabolic process, small-molecule catabolic process, organic-acid catabolic process, carboxylic acid catabolic process, and cofactor metabolic process pathways (Figure 1D). The differentially expressed genes were not enriched in the hepatic functionality-related pathways (Supplementary Figures S1C,D). Therefore, we concluded that there were no significant differences in liver function between different strains of rats and mice.

### Transcriptome Analysis Revealed that Liver-Function Genes in Mice and Human had Similar Expression

**FIGURE 2:**
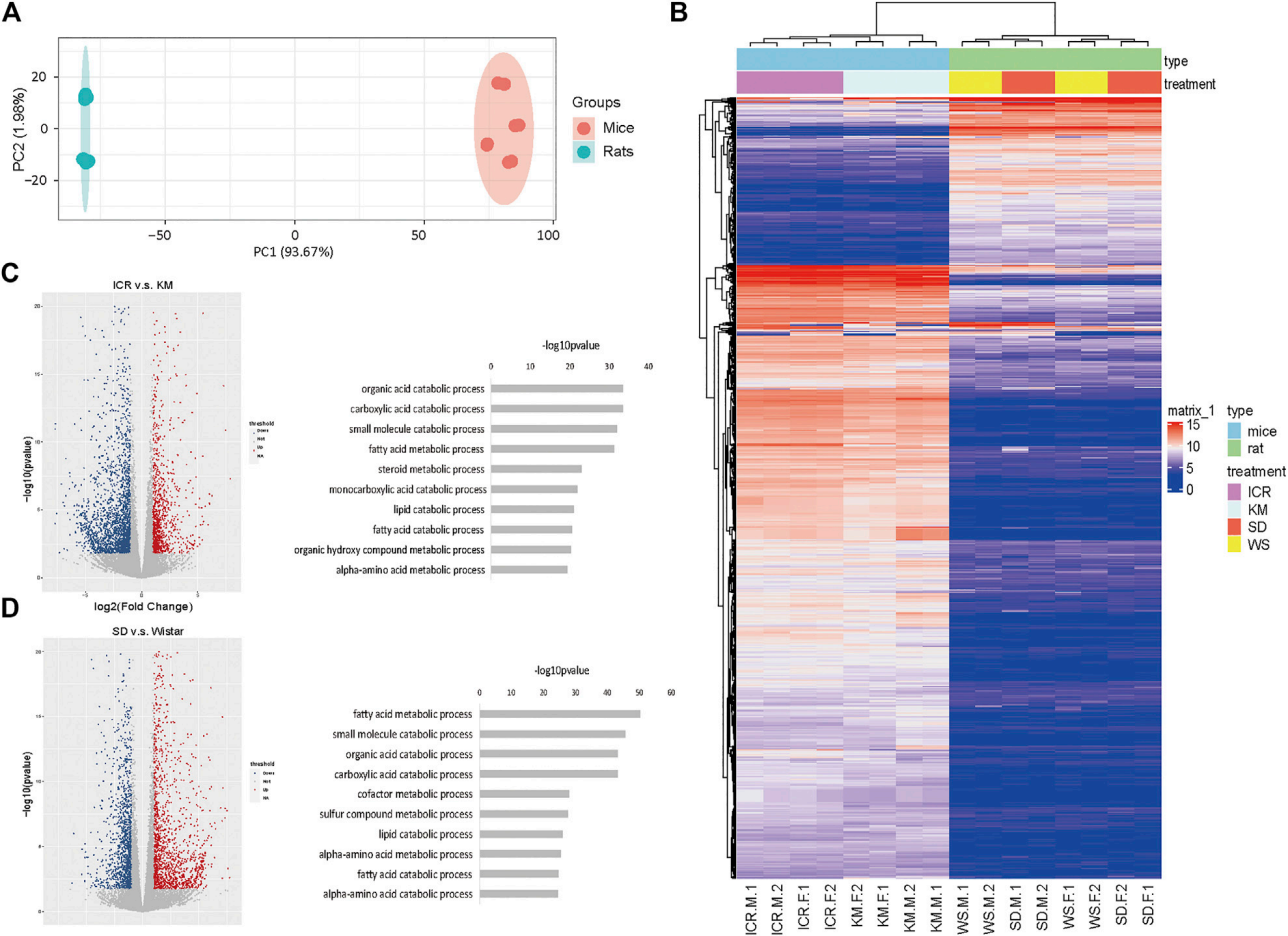
Mice and human liver function genes were more similar in expression **(A)** PCA profile of the expression of homologous genes in mice, rats, and humans. **(B)** Volcanic map showing the upregulated genes in differentially expressed genes of mice compared to rats; **(C)** The top enriched GO terms of RNA-seq from the differentially expressed genes; **(D)** GSEA of homologous genes of mice and rats; **(E)** The top 200 genes enriched GO pathway terms of RNA-seq from the homologous genes of human, rats, and mice; **(F)** RNA-Seq data of three cases of human liver from young adults downloaded and analyzed using the available literature; the homologous genes of humans, rats, and mice were selected to obtain the expression matrix, and the correlation map of the genes in the first 3,000 expression levels; **(G)** Dot plot of correlation value in humans, rats, and mice.

Next, we integrated the transcriptome data of rat, mice and hunman liver transcriptome data from database, there were significant differences between species in terms of overall gene expression (Figure 2A), suggesting that the selection of these genes was reasonable. Then we analyzed the expression of liver function more intimately. We selected the homologous genes from mice and rats for differential expression gene analysis (Figure 2B). The high expression genes enriched KEGG pathways in mice were enriched in fatty-acid elongation, sulfur metabolism, biosynthesis of unsaturated fatty acids, and ribosome and primary bile-acid biosynthesis (Figure 2C left) compared to that in rats. These results demonstrated that the genes related to lipid metabolism are generally highly expressed in mice. The low expression genes enriched KEGG pathways in mice were enriched in glutathione metabolism, viral protein interaction with cytokine and cytokine receptors, steroid biosynthesis, metabolism of xenobiotics by cytochrome P450, and linoleic acid metabolism (Figure 2C right) compared to that in rats. However, immune-related receptors, and metabolic enzyme-related genes was higher in rats compared to that in mice. The GO enrichment pathway is shown in Supplementary Figure S2A. Next, we performed GSEA on the homologous genes of mice and rats, and all of these genes were found to be related to liver function (Figure 2D and Supplementary Figure S2B). To clarify the differences in transcriptome expression profiles among rat, mouse, and human genera, we downloaded and analyzed three cases of RNA-Seq data of the human liver of young adults of the same age from the available literature (Cardoso-Moreira et al., 2019). By analyzing the homologous genes of the three species, we determined the GO (Figure 2E and Supplementary Figure S2C) and KEGG pathway (Supplementary Figure S2D) enrichment of the homologous genes based on the top 200 expression values of human, rats, and mice (Supplementary Table S2). We analyzed the top ten enrichment pathways and found that they all were involved in different functions. This may also be the reason why the same genes in a certain species perform different functions. Furthermore, the cluster and heatmap were generated by selecting the homologous genes at the first 3,000 expression among the three genera and the results indicated that mice were more similar to humans. Human, rat, and mouse genes were used for expression matrix analysis, and the correlation map revealed similar results (Figure 2F). We further calculated the correlation as shown in Figure 2G. It’s attests that the expression profiles of these genes were highly correlated between humans and mice.

### Mice were more Similar to Humans in Terms of Oxidative Stress, and Rats were more Similar to Humans in Terms of Fat Metabolism

**FIGURE 3:**
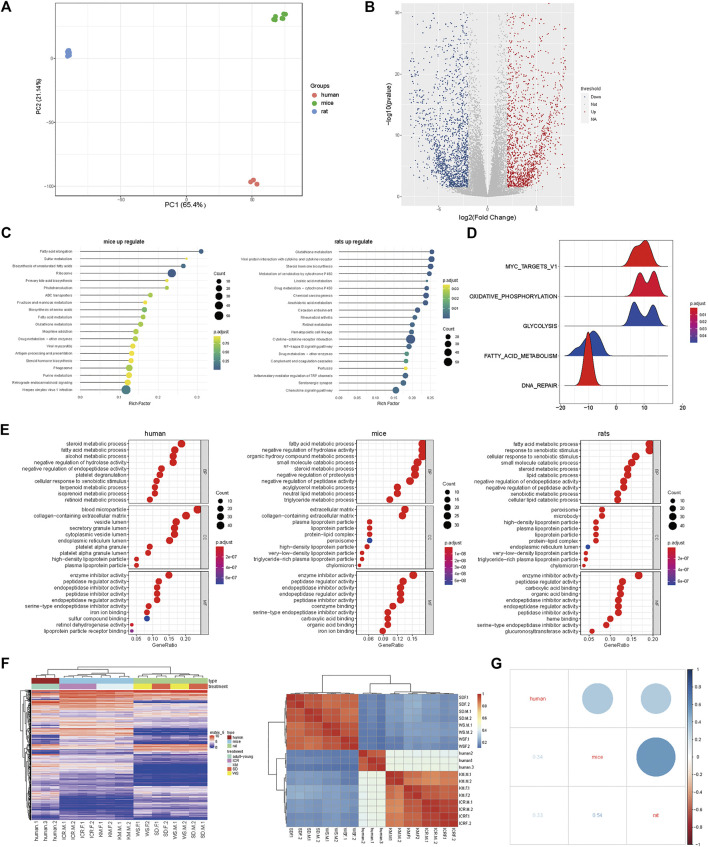
Mice were more similar to humans in terms of oxidative stress, and rats were more similar to humans in terms of fat metabolism. **(A)** Heatmap showing enrichment of oxidative stress in humans, mice, and rats; **(B)** Expression levels of three species in human oxidative stress gene set; **(C)** In the concentration of human oxidative stress genes, correlation with ICR mice **(left)**, and Kunming mice **(right)**. **(D)** Heatmap showing enrichment of steatosis in humans, rats, and mice; **(E)** Expression levels of three species using the human steatosis gene set **(left)**; **(F)** In the concentration of human steatosis genes, correlation with Sprague-Dawley Rats **(left)** and Wistar rats **(right)**.

We downloaded the human oxidative stress pathway gene set from the CTD (GO:0006979) database to determine the expression level of each species in the oxidative stress gene set. Our findings revealed higher expression in humans, followed by mice, and then rats (Figures 3A,B). We further analyzed the two mouse strains and found that Kunming mice had a higher correlation with humans compared with ICR mice. These results suggested that Kunming mice may be more suitable for the study of oxidative-stress related liver injuries (Figure 3C). In addition to liver-cell necrosis or apoptosis caused by oxidative stress, there is another type of liver injury that manifests as steatosis. Thus, we downloaded the human liver steatosis gene set from Human Phenotype Ontology (HP_0001397) and determined the expression level of each animal species in comparison with the human liver steatosis gene set. We found higher levels of expression in humans and rats. These results suggested that at the transcriptome level of hepatic steatosis, the expression in rats was more than that in mice (Figures 3D,E), suggesting that rats constitute a more suitable model to study liver steatosis-related diseases in humans. Next, we studied the expression of the two rat strains in comparison with the human liver steatosis gene set. Our results showed that compared to Wistar rats, SD rats constitute a more relevant model to study liver steatosis in humans (Figure 3F).

### Histopathological Studies and Gene Expression Confirmed that Kunming Mice were Better Suited for the Study of Oxidative Stress-Related DILI

**FIGURE 4:**
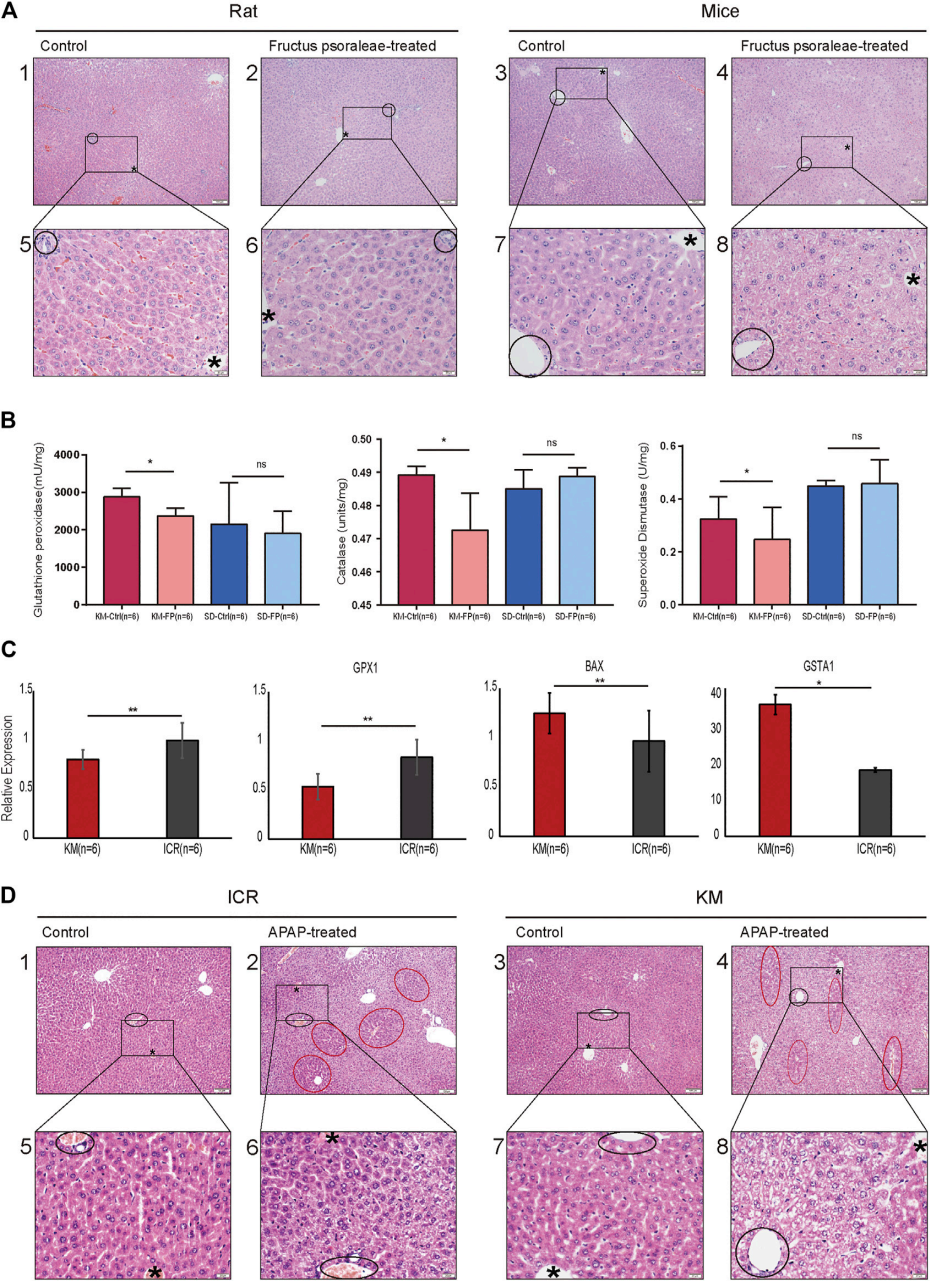
Histopathological studies and gene expression confirmed that Kunming mice were better suited for the study of oxidative stress-related DILI. **(A)** Liver HE section of rats and mice treated with the same dose of fructus psoraleae for 1 month. Scale bar: 1–4: 100 μm, 5–8: 20 μm, “*” is the hepatic lobular vein, black circle is the hepatic lobular portal area; **(B)** Glutathione peroxidase, catalase, and total superoxide dismutase assays in fructus psoraleae-treated Kunming mice and Sprague-Dawley rats. **(C)** Relative expression level of SOD1, GPX1, BAX, and GSTA1 in Kunming and ICR mice treated with the same dose (400 mg/kg) of APAP for 5 days; **(D)** Liver HE sections of Kunming and ICR mice treated with the same dose (400 mg/kg) of APAP for 5 days. Scale bar: 1–4: 100 μm, 5–8: 20 μm, “*” is the hepatic lobular vein, black circle is the hepatic lobular portal area, and red circle represents the region of normal hepatocytes.

Based on the above analysis, we chose fructus psoraleae, a traditional Chinese medicine known to cause oxidative stress-related DILI, to determine hepatotoxicity in mice and rats, respectively, using intragastric administration. Histopathological analysis of the liver tissue of mice and rats after treatment with fructus psoraleae revealed that there were different degrees of oxidative stress in mice and rats treated with this compound. No obvious abnormalities were found in the liver tissues of fructus psoraleae-treated rats (Figure 4A, left), whereas those of fructus psoraleae-treated mice showed diffuse hepatic vacuolar degeneration and cytoplasmic vacuity (Figure 4A, right). In addition, glutathione peroxidase activity in Kunming mice was lower than that in Sprague-Dawley rats after treatment with fructus psoraleae. Results from the catalase and total superoxide dismutase assays indicated that enzyme activities were significantly lower in fructus psoraleae-treated groups compared to the control groups in Kunming mice. Nearly no differences were observed in Sprague-Dawley rats between the treated and untreated groups (Figure 4B). We found that GSH levels were not significantly different in fructus psoraleae-treated groups compared to the control groups in Kunming mice and Sprague-Dawley rats; however, the data indicated a slight decrease in GSH levels in mice (Supplementary Figure S3A). These findings indicated that oxidative stress in fructus psoraleae-treated mice was more serious than that observed in rats, and was consistent with our previous analysis. To further determine the degree of oxidative stress in different mouse species, we reviewed the literature and found that APAP can induce oxidative stress in mice, which is significantly higher than that in rats. Therefore, the toxicity of APAP was tested in Kunming and ICR mice. Two days after gavage, one of the Kunming mice died. Pathological studies revealed that Kunming mice were more sensitive to APAP and died of APAP-induced hepatotoxicity. We verified the expression of oxidative stress-related genes in the two strains of mice treated with APAP. The relative mRNA expression of SOD1, and GPX1 in the APAP-treated group decreased in comparison with the control group. The expression level of the GPX1 gene, especially, was found to be decreased more in Kunming mice. CAT expression was not significantly different between Kunming and ICR mice; however, our findings showed a slight decrease in CAT in Kunming mice (Supplementary Figure S3B). Mitochondrial Bax translocation is an important and early mechanism to initiate DNA fragmentation and cell apoptosis. Our findings indicated that the mRNA and protein expression of Bax and GSTA1 were significantly increased in the APAP-treated groups compared to the control group. Moreover, the expression level was higher in Kunming mice (Figure 4C). Based on histopathological studies, we found that Kunming and ICR mice exhibited different degrees of oxidative stress. Both ICR and Kunming mice treated with APAP showed relatively obvious hepatocyte vacuolation and degeneration. The extent of degeneration in APAP-treated Kunming mice was greater than that in APAP-treated ICR mice (Figure 4D).

## Discussion and Conclusion

Based on the results obtained using transcriptome studies to explore the correlation between the hepatotoxicity phenotypes of two species of rats, two species of mice, and humans, we studied the biological pathways of the differences in hepatotoxicity among different species and identified the appropriate animal models that might be suitable for the study of different hepatotoxicity phenotypes.

From an evolutionary point of view, there must have been some differences among rats, mice, and humans. However, this aspect needs further analysis based on the study of the organs at the molecular level (Ohnishi et al., 2007; Zhao et al., 2020). First, we screened the homologous genes of rats and mice. After determining their significant correlation with liver function, the homologous genes were used to further analyze and compare the differentially expressed genes in mice and rats (Figures 2A,B). Subsequently, we downloaded human data to construct a principal component distribution map for the first 3,000 expression levels of genes. Our findings indicated that these 3,000 genes could be well distributed among species. Based on clinical data and literature reports, mainly two types of human liver injury were found to be well documented, namely, oxidative stress and hepatic steatosis (Giovanni et al., 2009). By referring to the literature and comparing the findings with the results generated in our study, we found that certain drugs, such as APAP, have different effects on mice and rats and even on different strains of mice and rats. Further, we downloaded the human gene set of oxidative stress and steatosis-related pathways from the database. The results indicated that the expression of oxidative stress-related genes in mice was higher than that in rats and was close to that in humans at the transcriptome level. These results suggested that a mouse model is more suited for the study of liver injury in humans caused by oxidative stress. Moreover, Kunming mice were found to have a higher correlation with humans compared to the correlation of ICR mice with humans, suggesting that the Kunming mouse model may be more suited for the study of liver injury, which results in oxidative stress in humans (Figures 3A–C). In the GSEA of steatosis, rats were found to have a higher expression of liver steatosis genes than mice. Thus, It could be inferred that rats are more suitable for the study of steatosis in humans as compared to mice. Meanwhile, the expression in the commonly used Sprague-Dawley and Wistar rats was studied in comparison with the human liver steatosis gene set. The correlation between Sprague-Dawley and humans was found to be higher compared to that with Wistar rats, suggesting that the former is more suitable for the study of liver injury caused by steatosis (Figures 3D–F).

Meanwhile, we used the traditional Chinese medicine, fructus psoraleae, which is known to cause hepatotoxicity in the form of oxidative stress in mice and rats (Wang et al., 2012; Cheung et al., 2009), as reported in our previous study. We found that fructus psoraleae-treated rats showed undetectable toxicity compared to that observed in mice (Figures 4A,B). To determine whether Kunming mice were more suited for the study of oxidative stress-related liver injury, we further analyzed the changes in oxidative stress-related genes at the mRNA level and performed histopathological studies using two strains of mice treated with APAP. Our findings revealed that Kunming mice were indeed well suited for the study of oxidative stress type liver injury (Figures 4C,D).

Our study has several limitations. First, we studied the differences among these species only at the transcriptome level. DNA and RNA methylation are important genetic mechanisms in mammalian development that play an important role in tissue differentiation and species development (Zhou et al., 2017). Therefore, it is important to further investigate the effect of modification of methylation in liver differentiation in the three species. Second, it is necessary to determine if gender plays a role in drug metabolism. In this study, the homologous genes of humans, rats, and mice were selected for expression matrix analysis (Figure 2C). Our findings revealed that gender differences in both rat strains and ICR mice were not obvious. However, we noted significant differences between male and female Kunming mice. The role of gender in alcohol-induced liver injury (Kono et al., 2000) and DILI (Sutti and Tacke 2018) have been reported in clinical studies. Thus, these findings warrant further follow-up studies. Lastly, additional experimental verification may be needed to further corroborate our findings. For example, other drugs that can cause oxidative stress and steatosis in different strains of mice should be further determined based on histopathological studies and the analysis of other pathways.

## Data Availability

The raw data supporting the conclusions of this article are uploaded to the Genome Sequence Archive in National Genomics Data Center (Zhang et al., 2020), Beijing Institute of Genomics (China National Center for Bioinformation) (https://bigd.big.ac.cn/gsa/) and can be accessed using the accession number CRA003625.

## References

[B1] Abzhanov A. (2013). von Baer's law for the ages: lost and found principles of developmental evolution. Trends Genet. 29 (12), 712–722. 10.1016/j.tig.2013.09.004 24120296

[B2] Breschi A. Gingeras T. R. Guigó R. (2017). Comparative transcriptomics in human and mouse. Nat. Rev. Genet. 18 (7), 425–440. 10.1038/nrg.2017.19 28479595 PMC6413734

[B3] Cardoso-Moreira M. Halbert J. Valloton D. Velten B. Chen C. Shao Y. (2019). Gene expression across mammalian organ development. Nature 571 (7766), 505–509. 10.1038/s41586-019-1338-5 31243369 PMC6658352

[B4] Chalasani N. P. Hayashi P. H. Bonkovsky H. L. Navarro V. J. Lee W. M. Fontana R. J. (2014). ACG clinical guideline: the diagnosis and management of idiosyncratic drug-induced liver injury. Am. J. Gastroenterol. 109 (7), 950–966. 10.1038/ajg.2014.131 24935270

[B5] Cheung W. L. Tse M. L. Ngan T. Lin J. Lee W. K. Tat W. P. (2009). Liver injury associated with the use of Fructus Psoraleae (Bol-gol-zhee or Bu-gu-zhi) and its related proprietary medicine. Clin Toxicol 47 (7), 683–5. 10.1080/15563650903059136 19640237

[B6] Costa W. Angelini C. De Feis I. Ciccodicola A. (2010). Uncovering the complexity of transcriptomes with RNA-seq. J. Biomed. Biotechnol. 2010, 853916. 10.1155/2010/853916 20625424 PMC2896904

[B7] Croucher N. J. Fookes M. C. Perkins T. T. Turner D. J. Marguerat S. B. Keane T. (2009). 'A simple method for directional transcriptome sequencing using Illumina technology. Nucleic Acids Res. 37 (22), e148. 10.1093/nar/gkp811 19815668 PMC2794173

[B8] Kalinka A. T. Tomancak P. (2012). The evolution of early animal embryos: conservation or divergence? Trends Ecol. Evol. 27 (7), 385–393. 10.1016/j.tree.2012.03.007 22520868

[B9] Kono H. Wheeler M. D. Rusyn I. Lin M. Seabra V. Rivera C. A. (2000). 'Gender differences in early alcohol-induced liver injury: role of CD14, NF-kappaB, and TNF-alpha. Am. J. Physiol. Gastrointest. Liver Physiol. 278 (4), 652–661. 10.1152/ajpgi.2000.278.4.g652 10762620

[B10] Liu L. W. Zhao X. Y. Jia J. D. (2019). [EASL clinical practice guidelines recommendations for drug-induced liver injury in 2019]. Zhonghua Gan Zang Bing Za Zhi 27 (6), 420–423. 10.3760/cma.j.issn.1007-3418.2019.06.006 31357756 PMC12769303

[B11] McGill M. R. Williams C. D. Xie Y. Ramachandran A. Jaeschke H. (2012). Acetaminophen-induced liver injury in rats and mice: comparison of protein adducts, mitochondrial dysfunction, and oxidative stress in the mechanism of toxicity. Toxicol. Appl. Pharmacol. 264 (3) 387–394. 10.1016/j.taap.2012.08.015 22980195 PMC3478469

[B12] Ohnishi T. Arnold L. L. Clark N. M. Wisecarver J. L. Cohen S. M. (2007). Comparison of endothelial cell proliferation in normal liver and adipose tissue in B6C3F1 mice, F344 rats, and humans. Toxicol. Pathol. 35 (7), 904–909. 10.1080/01926230701748081 18098037

[B13] Ray P. Torck A. Quigley L. Wangzhou A. Neiman M. Rao C. (2018). Comparative transcriptome profiling of the human and mouse dorsal root ganglia: an RNA-seq-based resource for pain and sensory neuroscience research. Pain 159 (7), 1325–1345. 10.1097/j.pain.0000000000001217 29561359 PMC6008200

[B14] Ren S. Peng Z. Mao J.-H. Yu Y. Yin C. Gao X. (2013). RNA-seq analysis of prostate cancer in the Chinese population identifies recurrent gene fusions, cancer-associated long noncoding RNAs and aberrant alternative splicings. Cell Res. 22 (5), 806–821. 10.1038/cr.2013.61 PMC3343650 22349460

[B15] Shen T. Liu Y. Jia S. Xie Q. Li J. Yan M. (2019). ' Incidence and etiology of drug-induced liver injury in mainland China. Gastroenterology. 156 (8), 2230-2241. 10.1053/j.gastro.2019.02.002 30742832

[B16] Straniero S. Laskar A. Savva C. Härdfeldt J. Angelin B. Rudling M. (2020). Of mice and men: murine bile acids explain species differences in the regulation of bile acid and cholesterol metabolism. J. Lipid Res. 61 (4), 480–491. 10.1194/jlr.ra119000307 32086245 PMC7112145

[B17] Sutti S. Tacke F. (2018). Liver inflammation and regeneration in drug-induced liver injury: sex matters!. Clin. Sci. 132 (5), 609–613. 10.1042/cs20171313 29545336

[B18] Tarantino G. Di Minno M. N. Capone D. Domenico C. (2009). Drug-induced liver injury: is it somehow foreseeable? World J. Gastroenterol. 15 (23), 2817–33. 10.3748/wjg.15.2817 19533803 PMC2698999

[B19] Touw I. P. Erkeland S. J. (2007). Retroviral insertion mutagenesis in mice as a comparative oncogenomics tool to identify disease genes in human leukemia. Mol. Ther. 15 (1), 13–19. 10.1038/sj.mt.6300040 17164770

[B20] Wang J.-B. Zhu Y. Bai Z.-F. Fu-Sheng W. Xiu-Hui L. (2018). Guidelines for the diagnosis and management of herb-induced liver injury. Chin. J. Integr. Med. 24 (9), 696–706. 10.1007/s11655-018-3000-8 29542018

[B21] Wang J. Jiang Z. Ji J. Li Y. Chen M. Wang Y. (2012). Evaluation of hepatotoxicity and cholestasis in rats treated with EtOH extract of Fructus Psoraleae. J. Ethnopharmacology 144 (1), 73–81. 10.1016/j.jep.2012.08.028 22954498

[B22] Wang Y. M. Yu C. L. (2014). 'Expert consensus on liver inflammation and its prevention. Chin. J. Pract. Intern. Med. 34 (02), 152–162. 10.1016/s0254-6272(14)60085-6

[B23] Wang Z. Gerstein M. Snyder M. (2009). RNA-Seq: a revolutionary tool for transcriptomics. Nat. Rev. Genet. 10 (1), 57–63. 10.1038/nrg2484 19015660 PMC2949280

[B24] Yang K. Woodhead J. L. Watkins P. B. Howell B. A. Brouwer K. L. R. (2014). Systems Pharmacology modeling predicts delayed presentation and species differences in bile acid-mediated troglitazone hepatotoxicity. Clin. Pharmacol. Ther. 96 (5), 589–598. 10.1038/clpt.2014.158 25068506 PMC4480860

[B25] Yu C. L. Mao Y. M. Chen C. W. (2015). 'Guidelines for the diagnosis and treatment of drug-induced liver injury. J. Clin. Hepatobiliary Dis. 31 (11), 1752–1769. 10.1007/s12072-017-9793-2

[B26] Zhang Z. Ma L. Abbasi A. A. Zehra Raza R. Gao F. (2020). Database resources of the national genomics data center in 2020.Nuclc Acids Res. 48, D24–D33. 10.1093/nar/gkz913 PMC7145560 31702008

[B27] Zhao F. B. Cheng L. Shao Q. Chen Z. X. Lv X. F. Li J. (2020). Characterization of serum small extracellular vesicles and their small RNA contents across humans, rats, and mice. Scientific Rep. 10 (1), 4197. 10.1038/s41598-020-61098-9 PMC7060188 32144372

[B28] Zheng H. Pomyen Y. Hernandez M. O. Li C. Livak F. Tang W. (2018). Single-cell analysis reveals cancer stem cell heterogeneity in hepatocellular carcinoma. Hepatology 68 (1), 127–140. 10.1002/hep.29778 29315726 PMC6033650

[B29] Zhou J. Sears R. L. Xing X. Y. Zhang B. Li D. F. Rockweiler N. B. (2017). 'Tissue-specific DNA methylation is conserved across human, mouse, and rat, and driven by primary sequence conservation. Bmc Genomics 18 (1), 724. 10.1186/s12864-017-4115-6 28899353 PMC5596466

