# Supplementary material for: Research on the Species Difference of the Hepatotoxicity of Medicine Based on Transcriptome
Source: Front Pharmacol. 2021 Apr 23;12:647084. doi: 10.3389/fphar.2021.647084 (PMC8115263; doi:10.3389/fphar.2021.647084)
Supplement: Supplementary file 2 [file Image3.tif]

Frontiers | Research on the Species difference of the hepatotoxicity of medicine based on transcriptome | Pharmacology


- About
- Journals
- Research Topics
- Articles
- More

Submit

My Frontiers

Office

- TSOF
  - TSOF
  - Article Production

Typesetter 3

frontiersproduction@tnq.co.in

- Profile
- Settings & Privacy
- Help Center
- Logout

Submit

**Impact Factor 4.225** | **CiteScore 5.0**More on impact ›

|  |  |
| --- | --- |
| Frontiers in Pharmacology | Gastrointestinal and Hepatic Pharmacology |

Toggle navigation


Section


- (current)Section
- About
- Articles
- Research topics
- For authors 
  - Why submit?
  - Fees
  - Article types
  - Author guidelines
  - Review guidelines
  - Submission checklist
  - Contact editorial office
  - Submit your manuscript
- Editorial board

- *Article alerts*

Articles


**Suggest a Research Topic >**

- 58
  total views

 View Article Impact

**Suggest a Research Topic >**

##### SHARE ON

- Facebook

  0
- Twitter

  0
- LinkedIn

  0
- AddThis

  New


## Original Research ARTICLE

Front. Pharmacol.
| doi: 10.3389/fphar.2021.647084

# Research on the Species difference of the hepatotoxicity of medicine based on transcriptome Provisionally accepted The final, formatted version of the article will be published soon. **Notify me**

Ziying Xu1,  Qianjun Kang1, Zihui Yu2, 3, 4, 
Lichun Tian1, 
 Jingxuan Zhang1\* and Ting Wang1\*

- 1Research Institute of Chinese Medicine, Beijing University of Chinese Medicine, China
- 2National Genomics Data Center, Beijing Institute of Genomics (CAS), China
- 3University of Chinese Academy of Sciences, China
- 4Key Laboratory of Genome and Precision Medicine, Beijing Institute of Genomics (CAS), China

In recent years, many drugs have been withdrawn from the market by drug oversight agencies due to hepatotoxicity, which makes the research of drug induced liver injury (DILI) more and more concerned. Most of the research on DILI tend to choose rats or mice as animal model for drug toxicity detection, but the toxicities of the same kind of drugs are often different in rats or mice. Such inconsistency of animal experiment results from different species of animals will affect the extrapolation of experimental results in human. So, it is particularly important to choose the most suitable animal model for drug hepatotoxicity research because there are great differences in genome through the evolution between rats and mice. In our research, genome wide transcriptome analysis was used to explore the liver toxicity caused by species difference. It will provide the preclinical basis for the research of drug hepatotoxicity mechanism and the selection of animal models for safety evaluation. The common rat and mice models (Sprague-Dawley rat and Wistar rat, ICR mice and Kunming mice) were used and by transcriptome sequencing, with the differentially expressed genes in rat and mouse livers as the entry point, we deeply explored the mechanism of oxidative stress and the difference of gene expression in fat metabolism pathway between rats and mice. Meanwhile, the clinical identified hepatotoxic drugs, fructus psoraleae and acetaminophen were used for validation, though pathology we confirmed that oxidative stress in mice is more serious than that in rats, and KM mice were more suitable for the study of oxidative stress-related drug induced liver injury. and the reliability of the results was further verified by gene expression. Our study will provide reference for the preclinical potential hepatotoxicity evaluation, prediction and early diagnosis of drug-induced liver injury caused by traditional Chinese medicine or Chemical drugs, and provide relevant research ideas for drug toxicity research.

Keywords: 
Hepatotoxicity, Drug induced liver injury, Species difference, Oxidative Stress, steatosis

Received: 29 Dec 2020;
Accepted: 08 Mar 2021.

Copyright: © 2021 Xu, Kang, Yu, Tian, Zhang and Wang. This is an open-access article distributed under the terms of the Creative Commons Attribution License (CC BY). The use, distribution or reproduction in other forums is permitted, provided the original author(s) and the copyright owner(s) are credited and that the original publication in this journal is cited, in accordance with accepted academic practice. No use, distribution or reproduction is permitted which does not comply with these terms.

\* Correspondence: 
  
 Prof. Jingxuan Zhang, Research Institute of Chinese Medicine, Beijing University of Chinese Medicine, Beijing, 100029, China, zh\_xyj@126.com   
 Prof. Ting Wang, Research Institute of Chinese Medicine, Beijing University of Chinese Medicine, Beijing, 100029, China, wangting1973@sina.com

Write a comment...

Add

##### COMMENTARY

##### ORIGINAL ARTICLE

##### People also looked at

## Pregnancy-Related Hormones Increase UGT1A1-Mediated Labetalol Metabolism in Human Hepatocytes

Raju Khatri, John K Fallon, Craig Sykes, Natasha Kulick, Rebecca JB Rementer, Taryn A Miner, Amanda P Schauer, Angela DM Kashuba, Kim A Boggess, Kim LR Brouwer, Philip C Smith and Craig R Lee

## Updates on Clinical use of Liquid Biopsy in Colorectal Cancer Screening, Diagnosis, Follow-up, and Treatment guidance

Omayma Mazouji, Abdelhak Ouhajjou, Roberto Incitti and Hicham Mansour

**Suggest a Research Topic >**

×

#### Supplementary Material

  

There is no supplementary material currently available for this article

Loading supplemental data...

  

|  | File Name |  |
| --- | --- | --- |
|  | Table 1.XLSX |  |
|  | Table 2.XLSX |  |
|  | Image 1.TIF |  |
|  | Image 2.TIF |  |
|  | Image 3.TIF |  |

  

Close

- About Frontiers
- Institutional Membership
- Books
- News
- Frontiers' social media
- Contact
- Careers
- Submit
- Newsletter
- Help Center
- Terms & Conditions
- Privacy Policy

© 2007 - 2021 Frontiers Media S.A. All Rights Reserved

### Privacy Preference Center

Our website uses cookies that are necessary for its operation. Additional cookies are only used with your consent. These cookies are used to store and access information such as the characteristics of your device as well as certain personal data (IP address, navigation usage, geolocation data) and we process them to analyse the traffic on our website in order to provide you a better user experience, evaluate the efficiency of our communications and to personalise content to your interests. Some cookies are placed by third-party companies with which we work to deliver relevant ads on social media and the internet. Click on the different categories' headings to change your cookie preferences. Click on "More Information" if you wish to learn more about how data is collected and shared.
More information

### Manage Consent Preferences

#### Strictly Necessary Cookies

Always Active

These cookies are necessary for the website to function and cannot be switched off in our systems. They are usually only set in response to actions made by you which amount to a request for services, such as setting your privacy preferences, logging in or filling in forms. You can set your browser to block or alert you about these cookies, but some parts of the site will not then work. These cookies do not store any personally identifiable information.

#### Analytics Cookies

Analytics Cookies

These cookies allow us to count visits and traffic sources so we can measure and improve the performance of our site. They help us analyse which pages are the most and least popular and see how visitors move around the site.    All information these cookies collect is aggregated and therefore anonymous.

#### Functional Cookies

Functional Cookies

These cookies enable the website to provide enhanced functionality and personalisation. They may be set by us or by third party providers whose services we have added to our pages. If you do not allow these cookies then some or all of these services may not function properly.

#### Advertising Cookies

Advertising Cookies

These cookies may be set through our site by our advertising partners. They may be used by those companies to build a profile of your interests and show you relevant adverts on other sites.    They do not store directly personal information, but are based on uniquely identifying your browser and internet device. If you do not allow these cookies, you will experience less targeted advertising.

### Back Button Performance Cookies

Vendor Search  Search Icon

Filter Icon

Clear

checkbox label label

Apply Cancel

Consent Leg.Interest

checkbox label label

checkbox label label

checkbox label label

Confirm My Choices
